# Piezo1-mediated fluid shear stress promotes OPG and inhibits RANKL via NOTCH3 in MLO-Y4 osteocytes

**DOI:** 10.1080/19336950.2022.2085379

**Published:** 2022-06-27

**Authors:** Zhongcheng Liu, Yuchen Tang, Liangzhi He, Bin Geng, Fan Lu, Jinwen He, Qiong Yi, Xuening Liu, Kun Zhang, Lifu Wang, Yayi Xia, Jin Jiang

**Affiliations:** Department of Orthopaedics, Gansu Key Laboratory of Orthopaedics, Lanzhou University Second Hospital, Lanzhou, Gansu, China

**Keywords:** Fluid shear stress, Piezo1, NOTCH3, RANKL, OPG, osteocyte

## Abstract

Piezo1, a mechanosensitive ion channel, participates in a variety of biological processes in maintaining bone homeostasis. As the most abundant cells in bones of the mammals, osteocytes play an essential role in bone formation, remodeling, and bone mass maintenance. Here, by exposing MLO-Y4 osteocytes to the fluid shear stress (FSS) microenvironment, we explored the effect of Piezo1-mediated FSS on the expression of the molecules critical to the process of bone formation and resorption, Receptor Activator of Nuclear Factor-Kappa-B Ligand (RANKL) and Osteoprotegerin (OPG). It was found that 9 dyne/cm^2^ loading for 30 minutes showed an upregulation trend on Piezo1 when MLO-Y4 osteocytes were exposed to an FSS microenvironment. FSS promotes the expression of OPG and inhibits the expression of RANKL. The blocker of Piezo1, GsMTx4, downregulates the effect of FSS on the expression of these two molecules. In addition, NOTCH3 was involved in this process. Thus, the results demonstrated that Piezo1-mediated FSS promotes the expression of OPG and inhibits the expression of RANKL via NOTCH3 in MLO-Y4 osteocytes.

## Introduction

As the most abundant cells in the bones of mammals, osteocytes play an essential role in bone formation, remodeling and bone mass maintenance [1]. Osteocytes are the major mechanosensory cells in bone tissue, which in turn exert the endocrine functions by sensing mechanical stimuli [2]. Mechanical stimuli have been identified as an important factor affecting cytokine secretion by osteocytes [3]. Fluid shear stress (FSS), a form of mechanical stimuli that exist in the extracellular matrix (ECM), is widely used to simulate mechanical stimuli in vitro [4,5]. Recently, the sense of FSS signals in osteocytes and how FSS regulates osteocytes have become the focus of current research.

Piezo1, a mechanosensitive ion channel, has been identified to play an important role in a variety of diseases, including blood pressure regulation [6], blood diseases [7], tumors [8], and bone diseases [9]. Existing literature has found that Piezo1 was activated by FSS, which indicated that Piezo1 is essential for bone metabolism [10,11]. Therefore, Piezo1-mediated FSS may be essential for bone homeostasis. Our previous study found that inhibition of Piezo1 down-regulated the migration of MC3T3-E1 osteoblasts [12]. Wang et al. found that the loss of Piezo1 in osteoblasts results in bone loss and bone resorption [13]. However, how Piezo1 regulates osteocytes still remains unclear.

Osteoprotegerin (OPG), since it was discovered by Simonet et al. [14], has been confirmed to have the role of bone protection [15]. The function of OPG was performed by inhibiting receptor activator of nuclear factor-κB ligand (RANKL), which promotes osteoclastogenesis [16]. Sojod et al. found that the overexpression of RANKL induces severe alveolar bone loss [17]. Deletion of RANKL results in increased bone mass [1]. RANKL was involved in the process of osteoclast formation, fusion, and activation [18]. Some pathways are found to be involved in the regulation of RANKL/OPG expression, including NOTCH3 [19], MAPK [20], TRPV4 [21], and Piezo1 [10]. As a newly discovered ion channel, how Piezo1 regulates the expression of RANKL and OPG remains unclear.

Therefore, this study aimed to explore the mechanism the effect of Piezo1-mediated FSS on RANKL/OPG via NOTCH3. We hypothesized that Piezo1-mediated FSS promotes OPG and inhibits RANKL via NOTCH3 in MLO-Y4 osteocytes.

## Materials and methods

### Detection of Piezo1, NOTCH3, RANKL/OPG in osteocytes

The analysis of single-cell transcriptomic data was performed by using the Single Cell Portal database (https://singlecell.broadinstitute.org), which stored the single-cell transcriptomic data from previous related studies. The expression of Piezo1, NOTCH3, and RANKL/OPG in bone marrow cells was analyzed by using the dataset from Zhong et al. [22] on the Single Cell Portal website.

### Cell culture

The MLO-Y4 cell line was kindly provided by Prof. Lynda Bonewald (University of Missouri-Kansas City, MO, USA). Mouse MLO-Y4 osteocyte-like cell line was cultured in α-minimum essential medium (Hyclone, USA) with 10% fetal bovine serum (Gibco, USA) and 100 u/mL penicillin-streptomycin at 37°C with 5%CO_2_.

### FSS loading

Cells were plated on 20*50 mm cover slides to load FSS. The α-minimum essential medium was used as a liquid environment for loading circulating FSS in parallel plate flow chambers as previously described [5]. In order to explore the influence of FSS on Piezo1, FSS with different time gradients (0, 15, 30, 45, 60, 90, 120 min) and different shear stress gradients (0, 3, 6, 9, 12, 15, 18 dyne/cm^2^) were set.

### Interventions on Piezo1 and NOTCH3

Yoda1 and GsMTx4 were used to activate and block Piezo1. Yoda1 (Selleck, China) was dissolved in the medium of MLO-Y4 cell line at the concentration of 10uM for 2 h as described by Li et al. [10]. GsMTx4 (Abcam) was dissolved in the medium of MLO-Y4 cell line at the concentration of 4uM for 0.5 h as described by Sun et al. [23]. Three siRNAs targeting the NOTCH3 signaling pathway were purchased from GenePharma (Shanghai, China). Reagent Lipofectamine 2000 (Invitrogen, USA) was used for transfection. Total RNA was extracted after 48 h transfection, and protein was extracted after 72 after transfection. The sequences are detailed in Table S1.

### Immunofluorescence analysis

After using 4% paraformaldehyde immobilization at room temperature, 0.1 Triton X-100 permeation at room temperature, and 10% goat serum block at 37°C for 30 min, respectively, Piezo1 antibody (15939-1-AP, Proteintech, 1:200) was used to incubate overnight at 4°C. Then CoraLite488-conjugated secondary antibody (SA00013-2, Proteintech, 1:300) was incubated for 1 hour and DAPI (Biosharp) for 20 min at 37°C. Pictures were taken under a fluorescence microscope (Olympus, Japan) with the same exposure time. Moreover, the individual cell was randomly grabbed in the images by image processing software Image-J (Version 1.52 V, National Institutes of Health, USA) to analyze the fluorescence intensity.

### qRT-PCR analysis

TRIzol reagent (Accurate Biotechnology, China) was used to isolate RNA of MLO-Y4 cells according to the manufacturer’s instructions. Evo M-MLV RT Kit (Accurate Biotechnology) was used to remove gDNA from cDNA and reverse transcription. Then, SYBR® Green Premix Pro Taq HS qPCR Kit (Accurate Biotechnology) was used to prepare amplification on LightCycler® 96 Instrument (Switzerland). GAPDH was used for normalization. The sequence is detailed in Tabled S1.

### Western blot analysis

Cells were treated with RIPA buffer (Beyotime Biotechnology, China) for lysing on ice for 30 min, and then centrifuged at 12, 000 rpm for 15 min at 4°C. After the supernatants were collected, the concentration of protein was detected by the BCA protein assay kit (Solarbio, China). Extracted protein was separated on 10% SDS-PAGE and then transferred onto PVDF membranes. After being blocked with 7.5% skimmed milk for 1 h, the membranes were incubated overnight at 4°C with the following antibodies, NOTCH3 (55114-1-AP, Proteintech, 1:300), RANKL (23408-1-AP, Proteintech, 1:300), Piezo1 (DF12083, Affinity, 1:1000), OPG (DF6824, Affinity, 1:1000), and β-actin (TA-09, ZSGB-BIO, 1:1500). Subsequently, the membranes were incubated with respective second antibodies (Proteintech) at 4°C for 1 h. The protein bands were observed on the ECL system (BioRad, USA) by the enhanced chemiluminescence method (Biosharp, China). Finally, the intensities of bands were quantified by using Image-J software.

### Statistical analysis

All experiments were independently repeated at least three times. The Kolmogorov-Smirnov test was used to test whether the distribution of the data was normal. Moreover, normally distributed quantitative data were presented as “mean ± standard deviation” (*SD*). In addition, statistical significance was calculated by using the two-tailed t test or one-way analysis of variance. *P*-value < 0.05 was regarded as a significant difference.

## Results

### Detection of Piezo1, NOTCH3, RANKL/OPG in osteocytes

**Figure 1.**
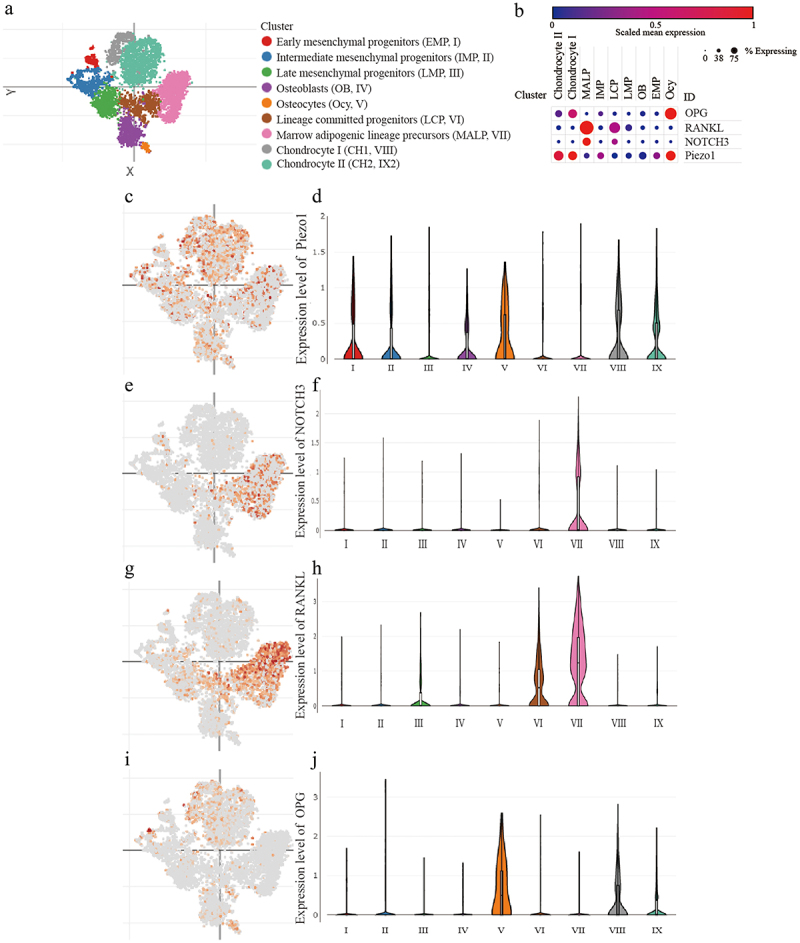
Detection of Piezo1, NOTCH3, RANKL/OPG in MLO-Y4 osteocytes a. The tSNE plot of the mesenchymal lineage cells; b. The bubble plot of the expression of Piezo1, NOTCH3, RANKL, and OPG among the mesenchymal lineage cells; c-d. The expression patterns of Piezo1 in tSNE plot and the violin plot; e-f. The expression patterns of NOTCH3 in tSNE plot and the violin plot; g-h. The expression patterns of RANKL in tSNE plot and the violin plot; i-j. The expression patterns of OPG in tSNE plot and the violin plot. Piezo1, Piezo Type Mechanosensitive Ion Channel Component 1; NOTCH3, Notch Receptor 3; RANKL, Receptor Activator of Nuclear Factor Kappa b Ligand; OPG, Osteoprotegerin; MALP, marrow adipogenic lineage precursor; IMP, intermediate mesenchymal progenitor; LCP, lineage committed progenitor; LMP, late mesenchymal progenitor; OB, osteoblast; EMP, early mesenchymal progenitor; Ocy, osteocyte.

According to the tSNE clustering of scRNA-seq data, all cells were divided into nine clusters (Figure 1(a)). Piezo1 was higher expressed in osteocyte, chondrocyte I (CH1), and chondrocyte II (CH2), in which the expression of OPG was relatively high but RANKL and NOTCH3 were relatively low. In contrast, Piezo1 was lower expressed in lineage-committed progenitors (LCP) and marrow adipogenic lineage precursors (MALP), in which the expression of OPG was relatively low but RANKL and NOTCH3 were relatively high. These results suggested that the expression of Piezo1 might be correlated with the expression of RANKL, OPG, and NOTCH3 (Figure 1).

### FSS up-regulates Piezo1 expression in MLO-Y4 osteocytes

**Figure 2.**
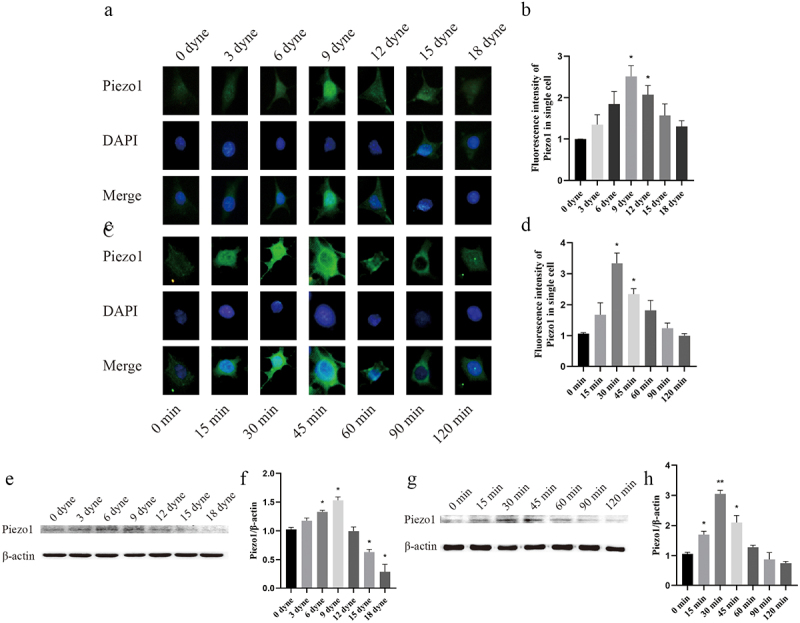
FSS up-regulates Piezo1 expression in MLO-Y4 osteocytes a. Piezo1 expression levels (green staining) were examined by fluorescence microscope with different FSS gradients.; b. Fluorescence intensity analysis of Piezo1 protein expression after treatment with different FSS gradients compared with control group, respectively; c. Piezo1 expression levels were examined by fluorescence microscope with different time gradients; d. Fluorescence intensity analysis of Piezo1 protein expression after treatment with different time gradients compared with control group, respectively; e. Effect of FSS on Piezo1 protein expression with different FSS gradients; f. The ratios of Piezo1/β-actin in different groups were quantified. g. Effect of FSS on Piezo1 protein expression with different time gradients; h. he ratios of Piezo1/β-actin in different groups were quantified. Data are shown as “mean ± SD” of at least three independent experiments. *P < 0.05; **P < 0.01.

To clarify the regulatory effect of FSS on Piezo1, MLO-Y4 cells were exposed to either time gradient (0, 15, 30, 45, 60, 90, 120 min) or strength gradient (0, 3, 6, 9, 12, 15, 18 dyne/cm^2^) FSS. Moreover, the expression level of Piezo1 was quantitatively analyzed by immunofluorescence intensity and western blot analysis. The results showed that when MLO-Y4 osteocyte was exposed to FSS in vitro, the expression of Piezo1 showed a unimodal tendency that firstly increased and then decreased with increasing FSS. Immunofluorescence intensity showed that Piezo1 was significantly activated at 9 dyne/cm^2^ (*P* = 0.0252), 12 dyne/cm^2^ (*P* = 0.0381), 30 min (*P* = 0.0212), and 45 min (*P* = 0.0224) when compared with the control group (Figure 2(a-d)). Western bolt analysis showed that Piezo1 was significantly activated at 6 dyne/cm^2^ (*P* = 0.0337), 9 dyne/cm^2^ (*P* = 0.0295), 15 min (*P* = 0.0100), 30 min (*P* = 0.0020), and 45 min (*P* = 0.0417) when compared with control group (Figure 2(e-h)). Thus, the load of 9 dyne/cm^2^ FSS for 30 min was selected to activate Piezo1 expression.

### Activation of Piezo1 inhibits the expression of NOTCH3 and RANKL, promotes the expression of OPG

**Figure 3.**
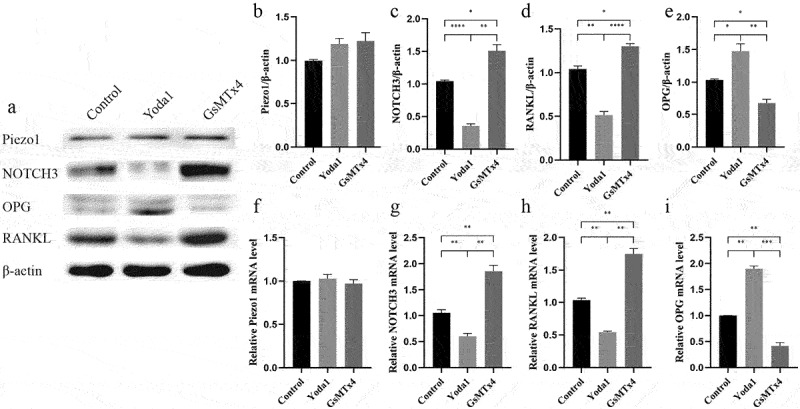
Activation of Piezo1 inhibits the expression of NOTCH3 and RANKL, promotes the expression of OPG MLO-Y4 cells were treated with Yoda1 (10uM) for 2 h, GsMTx4 (4uM) for 0.5 h. a. Western blot analysis of the protein expression levels of Piezo1, NOTCH3, RANKL, and OPG in MLO-Y4 cells; b-e. The ratios of Piezo1/β-actin, NOTCH3/β-actin, RANKL/β-actin, and OPG/β-actin in different groups were quantified; f-i. qRT-PCR analysis of mRNA expression levels of Piezo1, NOTCH3, RANKL, and OPG in MLO-Y4 cells. Data are shown as “mean ± SD” of at least three independent experiments. *P < 0.05, **P < 0.01, ***P < 0.001.

To investigate the influence of Piezo1 on NOTCH3, RANKL, and OPG, Piezo1 activator Yoda1 (10uM) and blocker GsMTx4 (4uM) was transferred to MLO-Y4 osteocyte for 2 h and 0.5 h, respectively. The expression level of NOTCH3, RANKL, and OPG were evaluated by qRT-PCR and western blot analysis. Yoda1 and GsMTx4 had no effect on Piezo1 protein (*P* > 0.05) and mRNA (*P* > 0.05) expression (Figure 3(a-f)). After Piezo1 was blocked by GsMTx4, the expression of NOTCH3 and RANKL increased while the expression of OPG decreased. On the contrary, after Piezo1 was activated by Yoda1, the expression of NOTCH3 and RANKL decreased while the expression of OPG increased. Western blot analysis showed that GsMTx4 significantly increased the expression of NOTCH3 (*P* = 0.0171) and RANKL (*P* = 0.0257) while Yoda1 significantly decreased NOTCH3 (*P* < 0.0001) and RANKL (*P* < 0.0080) expression (Figure 3(a-d)). GsMTx4 inhibited OPG expression (*P* = 0.0249) while Yoda1 promoted OPG expression (*P* = 0.0470) (Figure 3(a-e)). qRT-PCR showed that Yoda1 promoted OPG mRNA level (*P*= 0.0013) while GsMTx4 inhibited NOTCH3 and RANKL mRNA level (*P* = 0.0055, *P* = 0.0030, respectively) (Figure 3(g-i)).

### Piezo1-mediated FSS down-regulates the expression of NOTCH3 and RANKL, up-regulates the expression of OPG

**Figure 4.**
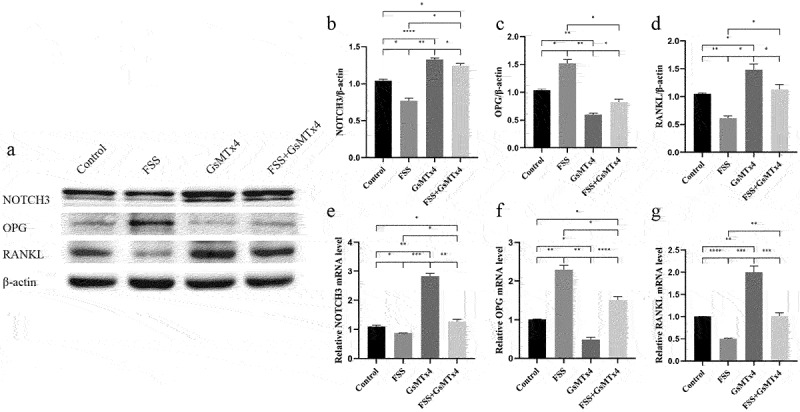
FSS-mediated Piezo1 down-regulates the expression of NOTCH3 and RANKL, up-regulates the expression of OPG MLO-Y4 cells were treated with FSS (9dyne/cm^2^, 30 min), GsMTx4 (4uM) for 0.5 h, and FSS + GsMTx4 (4uM) for 0.5 h. a. Western blot analysis of the protein expression levels of NOTCH3, RANKL, and OPG in MLO-Y4 cells; b-d. The ratios of NOTCH3/β-actin, OPG/β-actin, and RANKL/β-actin in different groups were quantified; e-g. qRT-PCR analysis of mRNA expression levels of NOTCH3, OPG, and RANKL in MLO-Y4 cells. Data are shown as “mean ± SD” of at least three independent experiments. *P < 0.05, **P < 0.01, ***P < 0.001, ****P < 0.0001.

To explore the regulatory role of Piezo1 in FSS mediated regulation of NOTCH3, RANKL, and OPG, four groups were set up: 1) Control; 2) FSS; 3) GsMTx4; 4) FSS+GsMTx4. FSS regulated the levels of NOTCH3, RANKL, and OPG through Piezo1. When Piezo1 was up-regulated by FSS, both the expression of protein and mRNA of NOTCH3 and RANKL were down-regulated subsequently, while expression of OPG protein and mRNA was up-regulated (Figure 4). Western blot analysis showed that FSS significantly reversed the blocking effect of Piezo1 by GsMTx4 and reduced the expression of NOTCH3 and RANKL (*P* = 0.0216, = 0.0224, respectively) (Figure 4(a-d)), FSS up-regulated the expression of OPG compared with the GsMTx4 group (*P* = 0.0251) (Figure 4(a-c)). qRT-PCR analysis showed a similar effect with the western blot on NOTCH3 (*P* = 0.0078), RANKL (*P* = 0.0004), and OPG (*P* < 0.0001) after Piezo1 was up-regulated by FSS compared with the GsMTx4 group (Figure 4(e-g)).

### Down-regulation of NOTCH3 inhibits the effect of GsMTx4-blocked Piezo1 on RANKL and OPG

**Figure 5.**
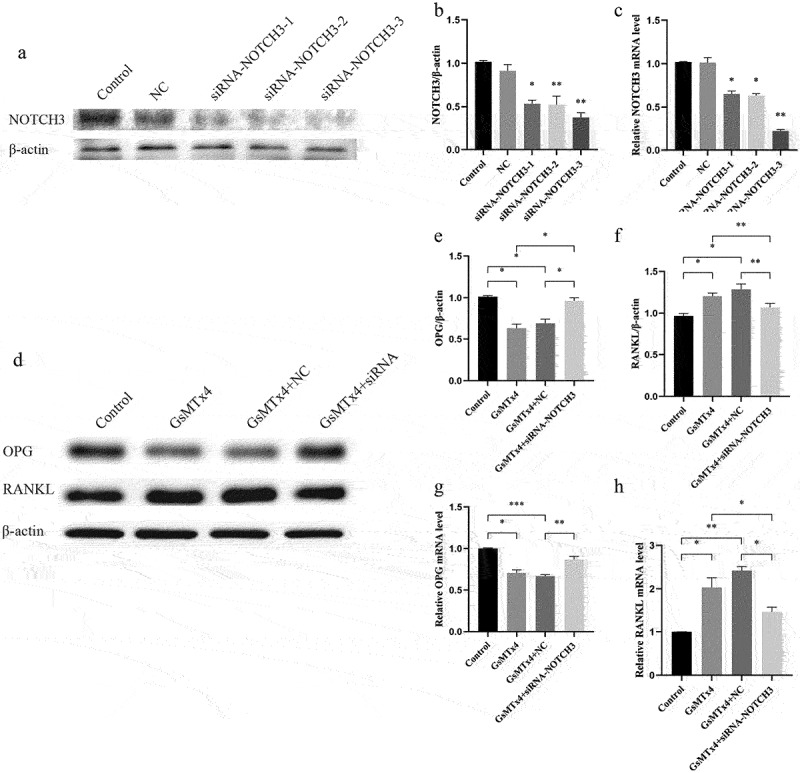
Down-regulation of NOTCH3 inhibits the effect of GsMTx4-blocked Piezo1 on RANKL and OPGA-C. MLO-Y4 cells were transfected with negative controls (NC), siRNA-NOTCH3-1, −2, or −3. a. Western blot analysis of the protein expression level of NOTCH3 in MLO-Y4 cells; b. The ratios of NOTCH3/β-actin in different groups were quantified; c. qRT-PCR analysis of mRNA expression levels of NOTCH3 in MLO-Y4 cells. d-h. MLO-Y4 cells were treated with GsMTx4 (4uM) for 0.5 h, GsMTx4 + NC, and GsMTx4 + siRNA-NOTCH3-3. e-f. The ratios of OPG/β-actin, and RANKL/β-actin in different groups were quantified; g-h. qRT-PCR analysis of mRNA expression levels of OPG, and RANKL in MLO-Y4 cells. Data are shown as “mean ± SD” of at least three independent experiments. *P < 0.05, **P < 0.01, ***P < 0.001.

To explore the role of NOTCH3 in the Piezo1 mediated expression of RANKL/OPG, small interference RNA was used to down-regulate NOTCH3 in the MLO-Y4 osteocyte. Three sequences of siRNA were synthesized. The effect of transfection reagent Lipofectamine 2000 on NOTCH3 was not statistically significant (Figure 5(a-c)). Compared with negative cells (NC) siRNA MLO-Y4 cells, the expression of NOTCH3 protein and mRNA in NOTCH3 siRNA-3 cells were declined by (58.7 ± 2.3)% and (79.3 ± 4.3)%, respectively. The transfection efficiency was the highest in siRNA-3 cells compared with NOTCH3 siRNA-1 and −2. Therefore, NOTCH3 siRNA-3 was selected for further experiments. After effectively down-regulating the expression of NOTCH3, GsMTx4 was used to block Piezo1 and the expression of RANKL and OPG were observed. Western blot analysis showed that NOTCH3 siRNA-3 significantly antagonized the GsMTx4-blocking effect on Piezo1, further upregulating the expression of OPG protein (*P* = 0.0314) (Figure 5(d,e)) and mRNA (*P* = 0.0091) (Figure 5(g)) as well as down-regulating that of RANKL protein (*P* = 0.0044) (Figure 5(d-f)) and mRNA (*P* = 0.0153) (Figure 5(h)).

## Discussion

According to our results, Piezo1 in MLO-Y4 osteocytes responds to the vitro FSS microenvironment. It is highly expressed and participates in the regulation of RANKL and OPG.We confirmed that Piezo1 mediated FSS promotes the expression of OPG and inhibits the expression of RANKL via NOTCH3. We further confirmed that down-regulation of NOTCH3 inhibits the effect of GsMTx4-blocked Piezo1 on RANKL and OPG.

The results of the present study demonstrated that Piezo1 responds rapidly to the appropriate intensity of FSS, especially 9 dyne/cm^2^ for 30 min. Piezo1 acts as a transmembrane mechanosensitive protein, which is expressed in a variety of mechanically sensitive cells with fluid flow in the surrounding environment [24]. Piezo1 opens and allows Ca^2+^ influs under the influence of mechanical stimuli, changing the membrane potential, which in turn translates mechanical stimuli into various signals affecting a variety of physiological and metabolic processes in cells [25]. Recently, some studies showed that Piezo1 is involved in the processes related to bone homeostasis. Our previous work firstly showed that Piezo1 was highly expressed in mouse MCT3-E1 osteoblasts and Piezo1 knocked down by siRNA can inhibit the migration ability of MC3T3-E1 cells [12]. Song et al. [11] found that Piezo1 is essential for osteoblast differentiation. The effect of mechanical signals on downstream molecules is blocked by silencing Piezo1 ion channel, which implies that the expression level of Piezo1 may be closely associated with abnormal bone metabolism. Li et al. [10] found that administration of Piezo1 agonist could increase the bone mass in adult mice, which indicates that the stimulation of Piezo1 by mechanical signals could effectively promotes bone metabolism.

We found that Yoda1 and GsMTx4 had no effect on the expression or Piezo1, but could affect the expression of downstream molecules, such as RANKL and OPG. Yoda1 [26] and GsMTx4 [27] work by opening and closing the channel structure of Piezo1. Ma et al. [28] concluded that Yoda1 and GsMTx4 had no effect on Piezo1 protein expression. Yoda1 and GsMTx4, as the activator and the blocker of Piezo1, did not affect the expression of Piezo1. These two reagents change the mechanical load sense of osteocytes by opening and closing Piezo1, and then affect the expression of molecules in osteocytes.

Our present study confirmed that Piezo1 is highly expressed in osteocytes. Over the past decades, osteocytes have emerged as mechano-sensors of bone and master regulators of bone homeostasis, through their control of osteoblast and osteoclast activities by the secretion of important regulatory factors. Sun et al. [23] extracted osteocytes from mice and confirmed that Piezo1 is required for bone formation. Therefore, the role of Piezo1 on osteocytes is very important for the ongoing process of bone formation, remodeling and maintenance of bone homeostasis.

**Figure 6.**
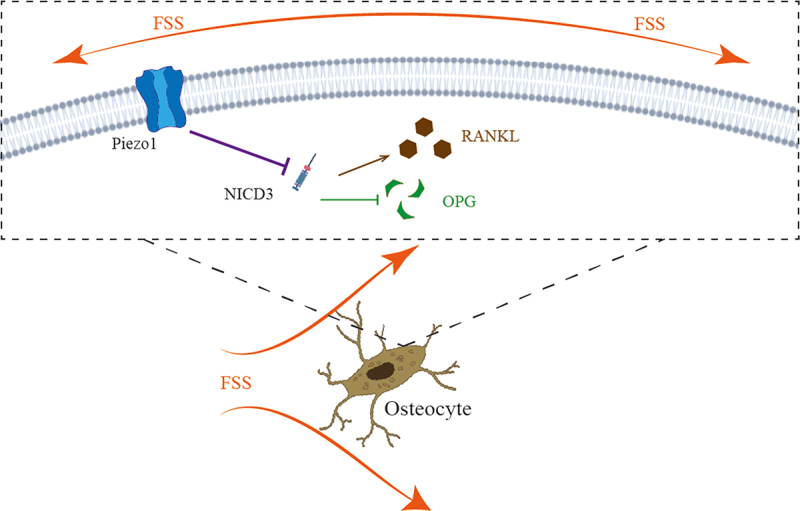
The schematic of the roles of how FSS-mediated Piezo1 ion channel regulates the expression of RANKL/OPG via NOTCH3 in osteocytes. FSS-mediated up-regulation of Piezo1 promotes the expression of OPG and inhibits the expression of RANKL via NOTCH3.

RANKL is a polypeptide expressed by osteoblasts [29] and osteocytes [30]. Deletion of RANKL results in increased bone mass [1]. OPG, a decoy receptor of RANK, has the function of inhibiting osteoclastogenesis [29]. RANKL/OPG plays an important role in the control of osteoclast biology. RANKL has been proved bone remodeling and osteoclast differentiation [1]. Li et al [10]. found that the expression of Tnfrsf11b (OPG) relative mRNA significantly decreased in Piezo1 knock-down MLO-Y4 cells, and FSS could significantly increase the relative mRNA expression of Piezo1. However, it was still unclear how Piezo1 regulates OPG. We added our results to current knowledge that Piezo1-mediated FSS could up-regulate the expression of OPG and down-regulate the expression of RANKL (Figure 6).

We further investigated the mechanism of Piezo1-mediated FSS regulates the expression of RANKL and OPG. NOTCH3, as a member of the NOTCH family, is a transmembrane signal receptor that releases NICD3 (NOTCH intracellular domain 3) into the cell after activation and participates in downstream physiological or pathological processes [31]. Canalis et al [19]. found that NOTCH3 induced Tnfsf11 (RANKL) relative mRNA expression and suppressed Tnfrsf11b in osteocytes. Thus, we performed this study to investigate whether Piezo1 could affect the expression of RANKL and OPG via NOTCH3. siRNA-NOTCH3 was used in this study. We confirmed that the expression of NOTCH3 could be inhibited after opening Piezo1 ion channels and that the expression of NOTCH3 could be down-regulated after FSS-induced up-regulation of Piezo1.

## Conclusion

In conclusion, FSS promotes the expression of OPG and inhibits the expression of RANKL. Piezo1 blocking using GsMTx4 blocked these effects. In addition, NOTCH3 was involved in this process. Thus, Piezo1-mediated FSS promotes the expression of OPG and inhibits the expression of RANKL via NOTCH3 in MLO-Y4 osteocytes.

## Supplementary Material

Supplemental Material

## Data Availability

The data that support the findings of this study are available from the corresponding author, [Y.X., or J.J.], upon reasonable request.
