# Supplementary material for: Piezo1-mediated fluid shear stress promotes OPG and inhibits RANKL via NOTCH3 in MLO-Y4 osteocytes
Source: Channels (Austin). 2022 Jun 27;16(1):127–36. doi: 10.1080/19336950.2022.2085379 (PMC9721416; doi:10.1080/19336950.2022.2085379)
Supplement: Supplemental Material [file KCHL_A_2085379_SM5469.zip › Supplemental File 1.docx]

Supplemental File 1. Sequences of the primers and siRNA

| Name | Sequence (5’ – 3’) |
| --- | --- |
| NOTCH3 (F) | CTTGGGAAATCTGCCTTACACT |
| NOTCH3 (R) | GTCCTTGTTGGCTCCATTTTTC |
| RANKL (F) | GGAAGCGTACCTACAGACTATC |
| RANKL (R) | AAAGTGGAATTCAGAATTGCCC |
| OPG (F) | ACCAGTGATGAGTGTGTGTATT |
| OPG (R) | AGAATTCGATCTCCAGGTAAGG |
| GAPDH (F) | TGTGTCCGTCGTGGATCTGA |
| GAPDH (R) | TTGCTGTTGAAGTCGCAGGAG |
| siRNA Negative control (sense) | UUCUCCGAACGUGUCACGUTT |
| siRNA Negative control (antisense) | ACGUGACACGUUCGGAGAATT |
| siRNA-NOTCH3-1 (sense) | CUGCCAAAGUGACAUAGAUTT |
| siRNA-NOTCH3-1 (antisense) | AUCUAUGUCACUUUGGCAGTT |
| siRNA-NOTCH3-2 (sense) | GGCUCAUUCUUGUGCCAAUTT |
| siRNA-NOTCH3-2 (antisense) | AUUGGCACAAGAAUGAGCCTT |
| siRNA-NOTCH3-3 (sense) | GGCCAGUUUACUUGCAUCUTT |
| siRNA-NOTCH3-3 (antisense) | AGAUGCAAGUAAACUGGCCTT |
