# Supplementary material for: Piezo1-mediated fluid shear stress promotes OPG and inhibits RANKL via NOTCH3 in MLO-Y4 osteocytes
Source: Channels (Austin). 2022 Jun 27;16(1):127–36. doi: 10.1080/19336950.2022.2085379 (PMC9721416; doi:10.1080/19336950.2022.2085379)
Supplement: Supplemental Material [file KCHL_A_2085379_SM5469.zip › Supplemental File 2.pdf]

Standard Material Transfer Agreement
For the Transfer of Biological Materialsbetween Non-profit Organizations

The Provider and Recipient identified below hereby agree to be bound by the terms set forth in the attached Exhibit A, and Exhibit B if applicable, to govern the transfer of the Original Material described herein. Each party represents that it has made no changes to the attached Exhibit A or Exhibit B as published by the Association of University Technology Managers and available on their website, except as modified by the checked boxes in Exhibit B.

☒ If checked, this Agreement is also subject to additional terms and conditions set forth on the attached Exhibit B. In the event of a conflict between any specific terms or conditions in Exhibit A and Exhibit B, Exhibit B shall govern.

**Provider** (the organization providing the Original Material)	**Recipient** (the organization receiving the Original Material)
Name: The University of Texas Health<br>Science Center at San Antonio<br>Address:7703 Floyd Curl Drive, MSC 7828<br>San Antonio, TX 78229-3900	Name: Orthopaedics Key Laboratory of Gansu Province<br>Lanzhou University<br>Address: No. 82 Cuiyingmen, Chengguan District<br>Lanzhou, Gansu<br>China

**Provider Scientist**	**Recipient Scientist**
Name: Lynda Bonewald, Ph.D.<br>Title: Professor	Name: ProfessorYayi Xia<br>Title: Director of Laboratory

**Original Material** (description of the material being transferred)	**Shipping Address**
MLO-Y4 osteocyte cell line	Name: Orthopaedics Key Laboratory of Gansu Province<br>Lanzhou University<br>Address:No. 82 Cuiyingmen, Chengguan District<br>Lanzhou, Gansu<br>China

Provider Authorized Signatory

Rachel E. Schofield
Signature

Rachel E. Schofield
Print Name

Manager, Contracts and AgreementsDirector of Laboratory
Title

24 September 2015
Date

Recipient Authorized Signatory

Signature

Professor Yayi Xia Yayi Xia
Print Name

Title

Date 2015-9-9

Recipient Scientist

Signature

Professor Yayi Xia Yayi Xia
Print Name

Date

Exhibit A
Standard Terms

I. DEFINITIONS:

1. **Provider**: Organization providing the Original Material. The name and address of this party is specified on page 1 of this Agreement.

2. **Provider Scientist**: The name and address of this party is specified on page 1 of this Agreement.

3. **Recipient**: Organization receiving the Original Material. The name and address of this party is specified on page 1 of this Agreement.

4. **Recipient Scientist**: The name and address of this party is specified on page 1 of this Agreement.

5. **Original Material**: The description of the Material being transferred is specified on page 1 of this Agreement.

6. **Material**: Original Material, Progeny, and Unmodified Derivatives. The Material shall not include:
   (a) Modifications, or (b) other substances created by the Recipient through the use of the Material which are not Modifications, Progeny, or Unmodified Derivatives.

7. **Progeny**: Unmodified descendant from the Material, such as virus from virus, cell from cell, or organism from organism.

8. **Unmodified Derivatives**: Substances created by the Recipient which constitute an unmodified functional subunit or product expressed by the Original Material. Some examples include: subclones of unmodified cell lines, purified or fractionated subsets of the Original Material, proteins expressed by DNA/RNA supplied by the Provider, or monoclonal antibodies secreted by a hybridoma cell line.

9. **Modifications**: Substances created by the Recipient which contain/incorporate the Material.

10. **Commercial Purposes**: The sale, lease, license, or other transfer of the Material or Modifications to a for-profit organization. Commercial Purposes shall also include uses of the Material or Modifications by any organization, including Recipient, to perform contract research, to produce or manufacture products for general sale, or to conduct research activities that result in any sale, lease, license, or transfer of the Material or Modifications to a for-profit organization. However, industrially sponsored academic research shall not be considered a use of the Material or Modifications for Commercial Purposes per se, unless any of the above conditions of this definition are met.

11. **Nonprofit Organization(s)**: A university or other institution of higher education or a not-for-profit organization officially recognized or qualified under the laws of the country in which it is organized or located, or any nonprofit scientific or educational organization qualified under a federal, state or local jurisdiction's nonprofit organization statute. As used herein, the term also includes national, state or local government agencies.

II. TERMS AND CONDITIONS OF THIS AGREEMENT:

1. The Provider retains ownership of the Material, including any Material contained or incorporated in Modifications.

2. The Recipient retains ownership of: (a) Modifications (except that, the Provider retains ownership rights to the Material included therein), and (b) those substances created through the use of the Material or Modifications, but which are not Progeny, Unmodified Derivatives or Modifications (i.e., do not contain the Original Material, Progeny, Unmodified Derivatives). If either 2 (a) or 2 (b) results from the collaborative efforts of the Provider and the Recipient, joint ownership may be negotiated.

3. The Recipient and the Recipient Scientist agree that the Material:

   (a) is to be used solely for teaching and academic research purposes;

   (b) will not be used in human subjects, in clinical trials, or for diagnostic purposes involving human subjects without the written consent of the Provider;

   (c) is to be used only at the Recipient organization and only in the Recipient Scientist laboratory under the direction of the Recipient Scientist or others working under his/her direct supervision; and

   (d) will not be transferred to anyone else within the Recipient organization without the prior written consent of the Provider.

4. The Recipient and the Recipient Scientist agree to refer to the Provider any request for the Material from anyone other than those persons working under the Recipient Scientist's direct supervision. To the extent supplies are available, the Provider or the Provider Scientist agrees to make the Material available, under an agreement having terms consistent with the terms of this Agreement, to other scientists (at least those at Nonprofit Organization(s)) who wish to replicate the Recipient Scientist's research; provided that such other scientists reimburse the Provider for any costs relating to the preparation and distribution of the Material.

5. (a) The Recipient and/or the Recipient Scientist shall have the right, without restriction, to distribute substances created by the Recipient through the use of the Original Material only if those substances are not Progeny, Unmodified Derivatives, or Modifications.

(b) Under an agreement at least as protective of the Provider's rights as this Agreement, the Recipient may distribute Modifications to Nonprofit Organization(s) for research and teaching purposes only.

(c) Without written consent from the Provider, the Recipient and/or the Recipient Scientist may NOT provide Modifications for Commercial Purposes. It is recognized by the Recipient that such Commercial Purposes may require a commercial license from the Provider and the Provider has no obligation to grant a commercial license to its ownership interest in the Material incorporated in the Modifications. Nothing in this paragraph, however, shall prevent the Recipient from granting commercial licenses under the Recipient's intellectual property rights claiming such Modifications, or methods of their manufacture or their use.

6. The Recipient acknowledges that the Material is or may be the subject of a patent application. Except as provided in this Agreement, no express or implied licenses or other rights are provided to the Recipient under any patents, patent applications, trade secrets or other proprietary rights of the Provider, including any altered forms of the Material made by the Provider. In particular, no express or implied licenses or other rights are provided to use the Material, Modifications, or any related patents of the Provider for Commercial Purposes.

7. If the Recipient desires to use or license the Material or Modifications for Commercial Purposes, the Recipient agrees, in advance of such use, to negotiate in good faith with the Provider to establish the terms of a commercial license. It is understood by the Recipient that the Provider shall have no obligation to grant such a license to the Recipient, and may grant exclusive or non-exclusive commercial licenses to others, or sell or assign all or part of the rights in the Material to any third party(ies), subject to any pre-existing rights held by others.

8. The Recipient is free to file patent application(s) claiming inventions made by the Recipient through the use of the Material but agrees to notify the Provider upon filing a patent application claiming Modifications or method(s) of manufacture or use(s) of the Material.

9. Any Material delivered pursuant to this Agreement is understood to be experimental in nature and may have hazardous properties. THE PROVIDER MAKES NO REPRESENTATIONS AND EXTENDS NO WARRANTIES OF ANY KIND, EITHER EXPRESSED OR IMPLIED. THERE ARE NO EXPRESS OR IMPLIED WARRANTIES OF MERCHANTABILITY OR FITNESS FOR A PARTICULAR PURPOSE, OR THAT THE USE OF THE MATERIAL WILL NOT INFRINGE ANY PATENT, COPYRIGHT, TRADEMARK, OR OTHER PROPRIETARY RIGHTS.

10. Except to the extent prohibited by law, the Recipient assumes all liability for damages which may arise from its use, storage or disposal of the Material. The Provider will not be liable to the Recipient for any loss, claim or demand made by the Recipient, or made against the Recipient by any other party, due to or arising from the use of the Material by the Recipient, except to the extent permitted by law when caused by the gross negligence or willful misconduct of the Provider.

11. This Agreement shall not be interpreted to prevent or delay publication of research findings resulting from the use of the Material or the Modifications. The Recipient Scientist agrees to provide appropriate acknowledgement of the source of the Material in all publications.

12. The Recipient agrees to use the Material in compliance with all applicable statutes and governmental regulations and guidelines such as, for example, those relating to research involving the use of animals or recombinant DNA.

13. This Agreement will terminate on the earliest of the following dates: (a) on completion of the Recipient's current research with the Material, or (b) on thirty (30) days written notice by either party to the other, or (c) on the date specified in Exhibit B, provided that:

(i) if termination should occur under 13(a) or (c) above, the Recipient will discontinue its use of the Material and will, upon direction of the Provider, return or destroy any remaining Material. The Recipient, at its discretion, will also either destroy the Modifications or remain bound by the terms of this agreement as they apply to Modifications;

and

(ii) in the event the Provider terminates this Agreement under 13(b) other than for breach of this Agreement or for cause such as an imminent health risk or patent infringement, the Provider will defer the effective date of termination for a period of up to one year, upon request from the Recipient, to permit completion of research in progress. Upon the effective date of termination, or if requested, the deferred effective date of termination, Recipient will discontinue its use of the Material and will, upon direction of the Provider, return or destroy any remaining Material. The Recipient, at its discretion, will also either destroy the Modifications or remain bound by the terms of this agreement as they apply to Modifications.

14. Paragraphs 6, 9, and 10 shall survive termination.

Exhibit B
Optional Terms

If checked, the following terms apply to this Agreement:

☒ This Agreement shall terminate on December 31, 2019. Upon termination, the Recipient will either destroy any remaining Material or return it to the Provider, as directed by the Provider.

☐ A transmittal fee of $ shall be paid by Recipient to Provider, for preparation and distribution costs.

☒ The Recipient intends to use the Material for purposes including but not limited to those described below:
Investigation of the mechanism of osteocyte exposed to mechanical load.

☐ To the extent permitted by law, Recipient agrees to treat in confidence, for a period of three (3) years from the date of its disclosure, any of Provider's written information about the Material that is stamped "Confidential" ("Confidential Information"). Any oral disclosures from Provider to Recipient shall be identified as being Confidential Information by notice delivered to Recipient within ten (10) days after the date of the oral disclosure. Confidential Information does not include information that:

a. has been published or is otherwise publicly available at the time of disclosure to the Recipient;

b. was in the possession of or was readily available to the Recipient without being subject to a confidentiality obligation from another source prior to the disclosure;

c. has become publicly known, by publication or otherwise, not due to any unauthorized act of the Recipient;

d. Recipient can demonstrate it developed independently, or acquired without reference to or reliance upon Confidential Information; or

e. is required to be disclosed by law, regulation, or court order.

☐ Additional binding terms:
