# Supplementary material for: Piezo1-mediated fluid shear stress promotes OPG and inhibits RANKL via NOTCH3 in MLO-Y4 osteocytes
Source: Channels (Austin). 2022 Jun 27;16(1):127–36. doi: 10.1080/19336950.2022.2085379 (PMC9721416; doi:10.1080/19336950.2022.2085379)
Supplement: Supplemental Material [file KCHL_A_2085379_SM5469.zip › Data_Availability_Statement.docx]

Data Availability Statement

The data that support the findings of this study are available from the corresponding author, [Y.X.], upon reasonable request.

Yayi Xia, MD, PhD.

No. 82 Cuiyingmen, Chengguan District, Lanzhou, Gansu, China.

xiayylzu@126.com
